# Supplementary material for: Red Rice Bran Extract Attenuates Adipogenesis and Inflammation on White Adipose Tissues in High-Fat Diet-Induced Obese Mice
Source: Foods. 2022 Jun 24;11(13):1865. doi: 10.3390/foods11131865 (PMC9266166; doi:10.3390/foods11131865)
Supplement: Supplementary file 1 [file foods-11-01865-s001.zip › foods-1771472-supplementary.pdf]

Supplementary Figure S1

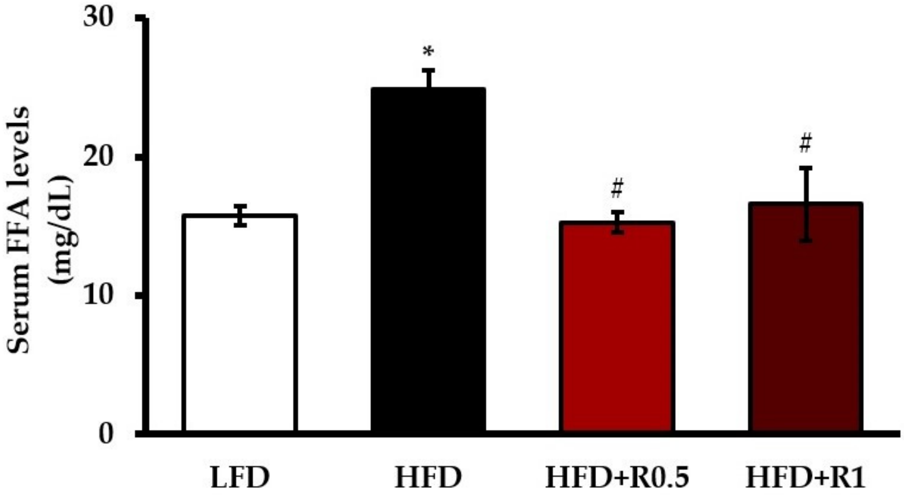

Figure S1. Effects of RRBE on serum FFA levels in HFD-fed mice. Data are presented as mean±SEM of 4 mice per group and analyzed by one-way ANOVA followed by Tukey's post hoc test. * $p < 0.05$ versus LFD group; # $p < 0.05$ versus HFD group.
